# The role of minimally invasive surgery in epithelial ovarian cancer treatment: a narrative review

**DOI:** 10.3389/fmed.2023.1196496

**Published:** 2023-06-14

**Authors:** Matteo Generali, Gianluca Annunziata, Debora Pirillo, Giovanni D’Ippolito, Gino Ciarlini, Lorenzo Aguzzoli, Vincenzo Dario Mandato

**Affiliations:** Unit of Obstetrics and Gynaecologic Oncology, Azienda USL-IRCCS di Reggio Emilia, Reggio Emilia, Italy

**Keywords:** minimal invasive surgery, diagnostic laparoscopy, early ovarian cancer, advanced ovarian cancer, interval debulking surgery, neoadjuvant chemotherapy, ovarian cancer recurrence, cytoreduction

## Abstract

**Objectives:**

The aim of this narrative review is to summarize the available evidence on the use of minimal invasive surgery (MIS) in the management of epithelial ovarian cancer (EOC).

**Background:**

MIS is currently performed to stage and treat EOC at different stage of presentation. We will evaluate risks and benefits of minimally invasive surgery for early stage EOC treatment, then potential advantages provided by staging laparoscopy in identifying patients suitable for primary cytoreductive surgery (PDS) will be discussed. Finally we will investigate the growing role of MIS in the treatment of advanced EOC after neoadjuvant chemotherapy (NACT) and in the treatment of EOC recurrence.

**Methods:**

An electronic database search was performed on PubMed, Medline, and Google Scholar for relevant studies up to December 2022.

**Conclusion:**

LPS represents a feasible surgical procedure for the staging and treatment in early, advanced and EOC relapse in selected patients treated in high-volume oncological centers by surgeons with adequate experience in advanced surgical procedures. Despite the increasing use of MIS over the last few years, randomized clinical trials are still needed to prove its effectiveness.

## Introduction

Epithelial ovarian cancer (EOC) affects approximately 225,000 women each year worldwide, and 140,000 die from this disease (1). EOC is usually diagnosed at an advanced stage because it is asymptomatic or has nonspecific symptoms that delay diagnosis (2). EOC usually affects old women, median age at diagnosis is 63 years old (2). The cornerstone of EOC treatment is surgery and adjuvant chemotherapy. EOC surgery requires multivisceral resection and several chemotherapies with a strong impact both on the body and on the emotional sphere of the patients because they upset the routine of EOC patients (3). Carboplatin and paclitaxel is the standard regimen in EOC patients, with a response rate of approximately 65%, median progression-free survival ranging from 16 to 21 months, and median overall survival ranging from 32 to 57 months (4). It is important to schedule surgery with a gynecologic oncologist in a referral centers to diagnose, stage, and treat this ominous disease (5). Many papers from different countries over the last 20 years have demonstrated how treatment at high volume referral centers can ensure cures associated with better oncological outcome (6, 7). High volume hospitals may ensure an interdisciplinary surgical approach and a multidisciplinary team that could manage EOC patients with a significant improvement in survival (8). Unfortunately, despite several evidences supporting centralization, not all EOC patients are treated in high-volume hospitals (9).

Minimally invasive surgery (MIS) in gynecologic oncology has widely expanded in the treatment of endometrial, cervical, and more recently in ovarian cancer (10). The term MIS refers to a wide variety of minimally invasive surgical approaches ranging from standard laparoscopy, robotics, mini-laparoscopy, and single-port laparoscopy. Based on patient characteristics, tumor extent, and type of surgery, surgeons choose the most appropriate method. The laparoscopic approach to adnexal masses started in the late 1970s (11) and it became more popular in gynecologic oncology after Daniel Dargent, Querleu and colleague described the first cases of laparoscopic pelvic lymphadenectomy in patients with cervical cancer and paraaortic lymph node dissection for carcinoma of the ovary or fallopian tube restaging (12–14).

By that time a growing numbers of data collected through many retrospective and prospective studies have been published to prove feasibility of laparoscopic surgery in the treatment of EOC in its different stages of presentation ranging from the treatment of early disease and staging procedures to the treatment of advanced EOC after neoadjuvant chemotherapy and in selected patients with recurrent tumors (15–17).

All those studies showed that in the past decade the indications for MIS in EOC staging and treatment have expanded beyond endometrial cancer staging to include surgical management of ovarian cancers. In this narrative review we will assess the role of laparoscopic surgery in treatment of early stage EOC (ESOC), the role of staging laparoscopy in surgical planning of primary debulking surgery, the role of laparoscopy in interval cytoreductive surgery of advanced ovarian cancer after neo adjuvant chemotherapy (NACT) and in recurrent EOC treatment.

## Materials and methods

A search of public databases (PubMed, MEDLINE, Google Scholar) until December 2022 was carried out to identify studies comparing the effectiveness of MIS in EOC treatment.

A string search was generated with the following medical subject: “minimal invasive surgery,” “diagnostic laparoscopy,” “early epithelial ovarian cancer,” “advanced ovarian cancer,” “interval debulking surgery,” “neoadjuvant chemotherapy,” “ovarian cancer recurrence,” “cytoreductive surgery.” All pertinent articles were retrieved, and the relative reference lists were reviewed in order to identify additional studies that could potentially be included. The shared criteria for considering a publication relevant were the design of the study, the sample size and the length of follow-up in studies reporting surgical data, the number of citations in other journals and in the case of publications performed by the same working group an attempt was made to consider the more recent publication. Non English language published literature, duplicates and abstract without full text have been excluded The publications found were equally divided and subjected to reading among the authors (MG, VDM, GA, DP, GD, GC, LA). In case of disagreements in the selection, a final decision was taken upon discussion with 2 authors (MG, VDM).

## Role of minimally invasive surgery in early ovarian cancer

ESOC only accounts for 20% to 25% of all EOC cases. Women with stage I have a 5-year survival rate of almost 90% as compared to 46% for women with advanced stages (1, 18). Most EOC are detected at an advanced stage since to date there are no tests or instrumental screening investigations applicable on a large scale to identify patients in the initial stage of the disease. As the development and validation of emerging biomarkers takes time, a renewed focus has been placed on improving clinical trial design in light of the failure of CA125-and Transvaginal ultrasound-based screening trials in order to identify approaches that can reduce EOC mortality efficiently (19). But in a context of daily clinical practice where laparoscopy is universally used for the treatment of ovarian pathology, ESOC is often detected during the removal of suspected benign ovarian tumors so a surgeon with the skills to perform a surgical-staging procedure might not be present.

Complete staging surgery for ESOC in fact includes hysterectomy, bilateral salpingo-oophorectomy, omentectomy, peritoneal biopsy, pelvic and para-aortic lymph node dissection, and peritoneal washings to identify occult advanced-stage disease (20, 21). Achieving accurate cancer staging is critical to predicting prognoses and to decide postsurgical treatment. The presence of microscopic metastases is found in up to 30% of women with apparent ESOC so obtaining prognostic information from a restaging procedure is essential in patients who did not undergo complete staging at the time of the initial surgery (22). Recent advances in technology and an increase in laparoscopic surgical expertise have resulted in the use of MIS to treat or restage ESOC worldwide (23). Laparoscopy guarantees advantages with better clinical outcomes in terms of less postoperative pain, less blood loss, shorter hospital day, and faster onset chemotherapy than laparotomy as previously published (24–26). As a result of the lack of randomized controlled trials (RCTs) and a lack of high-quality evidence, the Cochrane Collaboration concluded there was insufficient evidence to quantify the risks and benefits of laparoscopy when used for the treatment of ESOC (27). But considering the advantages associated with the laparoscopic approach even in the absence of RTC, the NCCN and ESMO-ESGO guidelines recommend a minimally invasive approach in the presence of gynecological oncological surgeons qualified in the staging and restaging of the EOC (5, 28). However main concerns remains about laparoscopic surgery disadvantages such as the potential rupture of the ovarian capsule, the risk of port site metastasis and the inability for lymph node manual assessment and palpation. Laparoscopy is in fact associated with a higher upstaging rate than laparotomy in the case of ovarian tumor rupture during surgery (24, 29–33). A study by Lee et al. revealed that tumor size was larger in the laparotomy group (29), this suggests the surgeon can select a surgical approach tailored to the ovarian neoplasia diameter to avoid undesired ruptures and in selected patients laparotomy and laparoscopy showed a similar incidence of tumor rupture as reported by Park (10.5% vs. 12.1% *p* = 1.000) (30). In case of intraoperative e tumor rupture, there is an immediate up staging, which may necessitate adjuvant chemotherapy and adversely affect the prognosis (34) but the rupture and not the surgical approach that can occur even during laparotomy seems to worsen the prognosis. The prognostic value needs to be investigated by RTC. Another potential disadvantage of MIS is the risk of port-site metastasis. There are a number of factors that can increase the risk of port-site metastases, including the presence of large masses in the abdomen and especially if concomitant ascites is present as reported in the Cochrane review (27). Some authors reported an incidence of port site metastases of up to 16% but in one of the largest detailed series reported by Zivancovic and by Rutten in 2008 and in 2017 the reported incidence was between 1.96 and 3% in line with other reports suggesting that port-site metastases in patients whose ovarian cancers are staged by laparoscopy may be rare (0%–2%) and only 1 case of the 20 port site metastases reported out of 1,694 patients undergoing laparoscopy did not present additional localization of disease in the abdomen (35–38). The correct staging of retroperitoneal disease may raise some concerns about MIS but meta-analysis has already shown for at least 10 years that no significant difference exists between MIS and traditional approaches when it comes to the size or number of lymph nodes removed during surgery (39). Lymph node metastases incidence in apparent early stage epithelial ovarian cancer is estimated to be around 14%–15% with approximately 37% of patients have only para-aortic positive nodes, 35% have only pelvic positive nodes, and the remaining 28% have both pelvic and para-aortic involvement. As stated by international guidelines a complete pelvic and para-aortic lymphadenectomy is recommended as part of surgical staging for ESOC and data on nodal status appear relevant to guide decisions on adjuvant therapy (40). However, the prognostic importance of the information provided by full nodal dissection must be balanced against the morbidity related to such a radical surgical procedure. Due to a low prevalence of nodal metastases in some histological subtypes (e.g., mucinous carcinoma of expansive subtype or low grade carcinoma), the indication for staging surgery in these cases may be questioned (28). Lymphatic mapping for the assessment of sentinel lymph nodes (SLNs) is a widely accepted part of the surgical treatment of breast and cutaneous melanoma and has been successfully implemented in several gynecological malignancies. The reported experience of SLN in ovarian cancer is restricted to a few studies with a small patient sample. Uccella et al. have recently published the largest prospective study (SELLY) on SLN in ovarian cancer with ICG alone as their laparoscopically tracer (41, 42). There is currently no recommendation from NCCN Clinical Practice Guidelines in Oncology 2022 edition (5) for application of SLN technique in ovarian cancer but there is growing support for its feasibility, and its acceptable negative predictive value. However, further evidence from phase III clinical studies is required to clarify the true negative predictive value, critically regarding patient safety. MIS safety has been examined in one of the largest multicenter studies currently available in the literature that included 300 patients with apparent ESOC referred to seven gynecologic oncology units for laparoscopic staging. In this study, MIS was found to have an advantage in terms of reducing morbidity and improving postoperative recovery. In addition, overall survival (OS), disease-free survival (DFS) and the rates of recurrence were similar to those reported in the literature for open surgical treatment of ESOC (43). Nevertheless, a recent systematic review and meta-analysis on retrospective or prospective data by Knisely et al. on patients with epithelial serous ovarian cancer who were treated with laparoscopic surgery indicates that laparoscopic surgery is a safe and technically feasible procedure (44). Even if data on survival and recurrence in patients staged by MIS show survival rates of approximately 90% at follow-up, similar to that observed in patients staged by laparotomy, these studies do not collect data on long term follow up as seen in Table 1. Studies of this size and duration are unlikely to be able to demonstrate the effectiveness of MIS in the EOC treatment (16, 23, 25, 30, 31, 45–50). In addition, MIS for staging and treatment of ESOC has not been the subject of a randomized controlled trial. For confirmation of these findings, and the development of selection criteria for MIS in ovarian cancer, large prospective randomized trial studies are required.

**Table 1:** Studies of laparoscopic surgical staging vs. open surgery for early ovarian cancer considering number of patients (*n*) tumor size, % of intraoperative spillage, lymph nodes removed, upstaging %, recurrence rate and survival outcome during follow up period.

Authors	Surgery (*n*)	Tumor size (cm)	Intraoperative spillage *N* (%)	Pelvic node n	PAN n	Upstaging *n* (%)	Mean time follow up months	Recurrence rate, *n* (%)	Survival outcome
Ghezzi et al. (16)	LPS 15	NA	3 (20)	25 ± 9.3	6.5	4 (26.7)	16	0	OS 100%
	LPT 19	NA	2 (10.5)	25.1 ± 5.8	7 ± 4.5	6 (31.6)	60	4 (7.1)	OS 100%
	P	-	0.63	0.96	0.78	1.0	-	-	-
Park et al. (29)	LPS 17	8.9 ± 6.3	2 (10.5)	27.2 ± 9.7	6.6 ± 6.2	5 (21.1)	17 (2–40)	2 (11.7)	OS 100%
	LPT 19	11 ± 6	4 (12.1)	33.9 ± 14.5	8.8 ± 8.1	7 (21.2)	23 (1–44)	0	OS 100%
	P	0.2	1.0	0.07	0.32	0.98	-	-	-
Lee et al. (24)	LPS 26	9.1 ± 5	0 (0)	23.5 ± 0.3	9.9 ± 7.4	1 (4)	12	0	PFS 100% OS 100%
	LPT 87	14 ± 8.3	13 (14.9)	22.8 ± 10.2	4.8 ± 4.1	5 (6)	25	0	PFS 91% OS 96.6%
	P	0.01	0.037	0.867	0.003		-	-	
Bogani et al. (45)	LPS 35	NA	6 (17)	22	10	15 (42)	64	4 (11)	NR
	LPT 32	NA	4 (12)	15	6	15 (46)	100	9 (28)	NR
	P	-	0.59	0.002	0.08		0.001	0.12	0.12 (5 years DFS) 0.26 (OS)
Koo et al. (46)	LPS 24	7.3 ± 4.3	13 (54.3)	26.8 ± 8.5	17.7 ± 10	NA	31.7	2 (8.3)	Mean DFS 59
	LPT 53	11.2 ± 4.5	21 (39.6)	27.8 ± 11.2	21.2 ± 11.2	NA	31.1	2 (3.8)	Mean DFS 66
	P	0.001	-	0.46	0.12	-	0.9	0.5	
Minig et al. (31)	LPS 50	6.75 (5–10)	0 (0)	15	10	12 (24)	25.9 (11.2–38.5)	6 (12)	PFS 73.6 months OS 85.4 months
	LPT 58	10 (7.7–15.2)	0 (0)	12	10	8 (14)	34.3 (28.4–47.8)	7 (12)	PFS 64.8 months OS 67 months
	P	0.001	-	-	-	0.173	0.004	0.785	0.63(PFS) 0.42(OS)
Gallotta et al. (23)	LPS 60	NA	NA	Total 16	LN (2–50)	NA	38 (24–48)	5 (8.3)	4 years PFS 89% OS 92%
	LPT 120	NA	NA	Total 18	LN (3–65)	NA	38 (24–48)	16 (13.3)	4 years PFS 81% OS 91%
	P	-	-	0.46	-	-		0.651	
Melamed et al. (47)	LPS 1,096	NR	NA	Total 14	LN (7–22)	133 (12)	28.7 (20.4–38.9)	NA	4 years OS 91.5%
	LPT 1,096	NR	NA	Total LN 12	LN (6–20)	210 (19)	29.3 (20.6–39.3)	NA	4 years OS 88.5%
	P	-	-	0.5		-	0.77	-	
Ditto et al. (48)	LPS 50	NA	NA	16.6 ± 7.9	16.7 ± 6.6	10 (20)	49.5	NA	-
	LPT 50	NA	NA	19.5 ± 9.3	18.4 ± 9.2	13 (26)	52.6	NA	-
	P	-	-	0.13	0.45	-	0.01	-	
Merlier et al. (49)	LPS 37	NR	0	12 (6–18)	7 (0–13)	NR	24 (11–50)	2 (5.4)	5 years OS 97.3%
	LPT 107	NR	4	14 (0–23)	9 (0–20)	NR	42 (22–66)	31 (28.9%)	5 years OS 79.8%
	P	-	0.24	0.026	0.27	-	<0.001	0.08	0.19
Wang et al. (50)	LPS 50	8 (6–12)	NA	26 (8–45)	5 (1–26)	5 (10%)	63.6 ± 27.6	7 (14%)	5 years OS 100% 5 years DFS 84.4%
	LPT 107	8 (6–11)	NA	26 (8–48)	7 (1–33)	13 (12.1%)	61.8 ± 28.2	8 (7.5%)	5 years OS 98.8% 5 years DFS 92.7%
	P	0.883	-	0.89	0.216	0.69	0.78		OS = 0.236 DFS = 0.242

LPS, Laparoscopy; LPT, Laparotomy; NA, Not assessed; NR, Not reported; PAN, Paraaortic lymph node; OS, overall survival; PFS, progression free survival; DFS, disease free survival.

## Role of laparoscopy in surgical planning of primary debulking surgery for ovarian cancer

Primary debulking surgery (PDS) is recommended as the standard surgical treatment for EOC (28) and optimal debulking surgery with removal of all visible disease remains the most significant prognostic factor for increasing survival in ovarian cancer patients (51, 52). Unfortunately, only about 30% of patients with ovarian cancer benefit from ultra-radical surgery with no visible residual tumor. In this context, it is imperative to identify patients with extensive disease who will likely have residual tumor after surgery despite being treated with radical multi-organ surgery with a moderate-high risk of complications and with no long-term survival benefits. The use of neoadjuvant chemotherapy (NACT) followed by interval debulking surgery (IDS), as an alternative strategy for such patients, has gained popularity over years (53–57). Studies suggest that this approach reduces surgical complexity and postoperative complications, especially in patients with a low performance status and/or high volume of disease (58–62), even if long term benefits remains controversial as a treatment option for EOC (57). While numerous efforts have been made to develop prediction models that integrate imaging techniques (CT, PET-TC), serum tumor markers, and clinical characteristics, they have been unable to accurately predict the effectiveness of optimal debulking (63). This results in the need to perform futile laparotomies, leading to possible intra and postoperative complications, an extended hospital stay, additional financial costs, and could cause a delay in initiating chemotherapy (25, 64, 65).

Starting from the experience reported by Vergote (66) on the use of staging laparoscopy (S-LPS) to guide the decision to perform or not perform PDS, the use of laparoscopy for advanced EOC surgery is increasingly being used as a tool to plan ultra-radical surgery and to reduce the number of futile laparotomies. Various retrospective and prospective studies had been published by that time (15, 66–70) and Fagotti et al. (71) developed an S-LPS-based quantitative model, see Table 2. Using an S-LPS predictive index value (PIV), they developed a simple scoring system to estimate the likelihood of optimal cytoreduction based on the presence of (I) omental cake, (II) peritoneal carcinomatosis, (III) diaphragmatic carcinomatosis, (IV) mesenteric retraction, (V) infiltration of the bowel and/or (VI) stomach, and (VII) liver metastases. Two points were assigned to each parameter if it was present. In patients with scores greater than 8, suboptimal surgery was predicted with a specificity of 100%, a positive predictive value of 100%, and a negative predictive value of 70%. With a PIV cut-off of 8, complete cytoreduction was 0%, whereas unnecessary laparotomies were 40.5% (15). A prospective multicenter trial (Olympia-MITO 13) conducted on 120 patients in four different centers aimed at evaluating if the PIV assessment was feasible and reproducible in external centers. Results published in 2013 showed that Fagotti index (PIV) reached an accuracy rate of 80% or greater and could be replicated in different centers with different expertise (72). A randomized controlled study published by Rutten et al. (36) showed that suboptimal PDS was estimated to have decreased from 39% to 10% using S-LPS, suggesting that S-LPS should be adopted as a standard clinical procedure.

**Table 2:** Fagotti laparoscopic predictive index value (PIV) score.

Laparoscopic feature	Score 0	Score 2
Peritoneal carcinomatosis	Carcinomatosis involving a limited area (along the paracolic gutter or the pelvic peritoneum) and surgically removable by peritonectomy	Unresectable massive peritoneal involvement with a miliary pattern of distribution
Diaphragmatic involvement	No infiltrating carcinomatosis and no nodules confluent with most of the diaphragmatic surface	Widespread infiltrating carcinomatosis or nodules confluent with most of the diaphragmatic surface
Mesenteric involvement	No large infiltrating nodules and no involvement of the root of the mesentery (i.e., movement of intestinal segments is not limited)	Large infiltrating nodules or involvement of the root of the mesentery indicated by limited movement of intestinal segments
Omental involvement	No tumor diffusion observed along the omentum up to the greater curvature of the stomach	Tumor diffusion observed along the omentum up to the greater curvature of the stomach
Bowel infiltration	No bowel resection assumed and no miliary carcinomatosis observed on the bowel ansae	Bowel resection assumed or miliary carcinomatosis observed on the ansae
Stomach infiltration	No obvious neoplastic involvement of the gastric wall	Obvious neoplastic involvement of the gastric wall
Liver metastases	No surface lesions	Any surface lesion

A value of 0 or 2 is assigned depending on whether disease is present in these locations. If patients score ≥ 10, optimal cytoreduction is very unlikely. If they score < 10, they are considered candidates for cytoreductive surgery.

The possible standardization of the S-LPS in determining disease resectability in patients with suspected advanced EOC and the numerous publications among different working groups allowed to evaluate the applicability of the technique through a Cochrane review recently published in 2019 (73). Analyses were performed on 18 studies involving 14 cohorts of patients. S-LPS had good overall accuracy but some women still had suboptimal resected disease (i.e., >1 cm residual tumor) at PDS. S-LPS, in fact, cannot be used to evaluate a substantial number of women. For example presence of adhesions may restrict access to the abdomen or prevent the exploration of the peritoneum in its entirety and S-LPS is incapable of assessing specific areas associated with suboptimal debulking (retroperitoneal, mesenteric, or retro-hepatic and peripancreatic region). Furthermore S-LPS is considered an easy and low-morbidity method of assessing advanced EOC patients but even if the complication rate for S-LPS is considered low (reported between 1% and 5%) the procedure still requires general anesthesia, and some complications have been reported as severe, thus potentially delaying the primary treatment (surgery or NACT) (70). Additionally, as previously described port-site metastases have been reported in up to 3% of cases following S-LPS, with ascites associated with an increased risk (74, 75). In conclusion, as often happens in the history of medicine regarding novel techniques applications, the absence of entirely univocal data and above all different surgical backgrounds have arisen a situation where S-LPS has motivated some institutions to include it as part of their standard diagnostic procedure, some others only perform S-LPS when there is doubt about resectability, while others do not perform S-LPS at all. Despite the personal affection or not to this technique S-LPS should be performed by oncological gynecologists minimizing the risk of complication and lowering the risk that less experienced gynecologists can send too many patients to NACT, in order to perform a potentially less demanding interval debulking surgery (9, 76). The focus in ovarian cancer referral centers should be on choosing the right surgical approach to treat patients with advanced-stage EOC, and aiming to improve surgical outcomes and the survival rate of patients by implementing systematic quality improvement initiatives.

## Minimally invasive interval cytoreductive surgery vs. laparotomy in the treatment of advanced ovarian cancer after NACT

Neoadjuvant chemotherapy has become increasingly popular worldwide as an alternative to primary cytoreductive surgery for advanced ovarian cancer. According to statistics from 2016, 1 in 3 patients with ovarian cancer stage IIIC or IV underwent chemotherapy before surgery (70). Women with high perioperative risk, or poor likelihood of achieving optimal cytoreduction during primary surgery, should receive neoadjuvant chemotherapy and interval surgery, according to guidelines developed by the American Society of Clinical Oncology and the Society of Gynaecologic Oncology (53). Women who have responded to neoadjuvant chemotherapy could benefit from minimally invasive interval cytoreduction as reported by small uncontrolled observational studies that shows better perioperative outcomes a high rate of complete cytoreduction and excellent progression-free survival rates (77–79). The National Cancer Database was utilized by Melamed et al. (80) in analyzing 450 women undergoing minimally invasive cytoreduction vs. 2,621 women undergoing laparotomy. It was found that both groups had similar overall survival rates and surgical outcomes, despite adjusting for several potential confounders. More recently, the INTERNATIONAL MISSION trial, a multi-center retrospective study demonstrated a median progression-free survival of 23 months and a 5-year overall survival rate of 52% in 127 women who underwent MIS after neoadjuvant chemotherapy for EOC with 96.1% patients with no residual tumor and 4.7% complication rate and a 3.9% conversion rate to laparotomy (81). Even though there are no randomized studies, the use of minimally invasive interval cytoreductive surgeries keeps rising (82) and the National Cancer Network Guidelines endorse the MIS as an approach for interval debulking surgery in “select patients” (83). Two meta-analyses have recently been published showing no deleterious survivals or recurrences associated with MIS for ovarian cancer. For women with advanced ovarian cancer who have responded to neoadjuvant chemotherapy, the current limited evidence suggests minimally invasive cytoreductive surgery is equivalent to open interval cytoreductive surgery at this time (44, 84). An ongoing randomized trial comparing these approaches will provide an assessment of the oncologic efficacy of MIS (85).

## Minimally invasive surgery for ovarian cancer recurrence

In approximately 75% of cases of EOC, recurrence occurs within 2 years from initial diagnosis (1). Recurrent ovarian cancer is typically treated with systemic chemotherapy and the effects of surgery, is still under debate due to the conflicting data obtained from retrospective reports that strongly support radical secondary cytoreduction effects of surgery (86–90). This data have been questioned by a large randomized trial promoted by the Gynaecologic Oncology Group (GOG-213), where secondary surgery did not improve overall survival, but only disease-free survival (91). Despite this, controversy surrounds the GOG-213 data due to the low rate of complete cytoreduction (63%). No matter what the debate on these topics is, surgical secondary cytoreduction (SSC) remains an important treatment option for ovarian cancer recurrence as suggested by ESMO-ESGO recommendation (28). MIS can have multiple indication in women with ovarian cancer recurrence. Considering that even in recurrence as well in naive patients a minimal invasive approach can be useful to assess the extent of disease and potential cytoreduction in addition to the already well-known benefits previously debated on intraoperative blood loss, transfusion risks, perioperative and postoperative complications, including a shorter time to initiation of adjuvant chemotherapy. The quality of evidence in ovarian cancer recurrence treatment by MIS is low, being based mainly on case reports, retrospective case series and retrospective comparative studies (92–101). In three studies comparing MIS vs. laparotomy the rate of optimal cytoreduction by minimally invasive secondary cytoreductive surgery is consistent across studies, ranging from 70% to 98% and no statistically significant differences on disease free and overall survival (92–94) Table 3. However, MIS requires a high level of expertise and skills, especially in this setting of patients, and should be performed in high-volume oncological centers with adequate experience in advanced surgical procedures. No guidelines or consistent data are currently available to identify patients eligible for MIS for recurrences and no predictors of its feasibility are currently available so the decision to undergo laparoscopic or robotic debulking is left to surgeons’ discretion in different studies. Most MIS candidates had single-site disease or few relapses. In order to confirm or not MIS, a diagnostic laparoscopy, together with preoperative imaging may be useful (102). In conclusion for the treatment of recurrent EOC, MIS may be deemed an alternative to laparotomy in highly selected cases at dedicated oncology centers and in the context of well-conducted research.

**Table 3:** Intraoperative and postoperative complication, complete cytoreduction rate (CCR = residual tumor = 0) and survival outcomes in EOC recurrence treated by minimally invasive surgery.

Reference	Intraoperative complications	Postoperative complications	CCR (R = 0)	Progression-free survival	Overall survival
Eriksson et al. (94)	MIS 0; LPT 0	MIS 3 (8%); LPT 15 (22%); (*p* = 0.06)	MIS 37 (95%); LPT 63 (93%); (P = 1.0)	2-year: MIS 56.1%; LPT 63.5%; (*p* = 1.0)	2-year: MIS 92.2%; LPT 81.4%; (P = 0.7)
Fagotti et al. (92)	MIS 0; LPT 1 (9.1%); (*p* = 0.92)	MIS 2 (18.2%); LPT 3 (27.3%); (*p* = 0.33)	MIS 11 (100%); LPT 11 (100%); (p = 1.00)	Not reported	Not reported
Magrina et al. (93)	LPS 2 (22.2%); LPT 7 (21.2%); Robot 1 (10%); (*p* = 0.77)	LPS 3 (33.3%); LPT 14 (42.4%); Robot 2 (20%); (*p* = 0.46)	LPS 8 (88.9%); LPT 24 (72.7%); Robot 7 (70%); (*p* = 0.66)	3-year: LPS 22.9%; LPT 33.1%; Robot 43.8%; (*p* = 0.95)	3-year: LPS 66.7%; LPT 48.4%; Robot 85.7%; (*p* = 0.18)
Total	MIS 3 (4.3%); LPT 8 (7.1%); (*p* = 0.58)	MIS 10 (14.5%); LPT 32 (28.6%); (*p* = 0.03)	MIS 63 (95.5%); LPT 98 (87.5%); (*p* = 0.81)	Not reported	Not reported

## Discussion

The cornerstone of EOC management is surgery and adjuvant chemotherapy. It is important to schedule surgery with a gynecologic oncologist in a referral center to diagnose, stage, and treat this disease (5–9). In the 1970s, MIS started to be used in gynecological malignant pathologies and during years the number of surgeons who thought MIS was appropriate for the treatment of endometrial, cervical an ovarian cancer increased significantly (10). The rate of serious complications associated with MIS is low. The most common complications are vessels and bowel injuries resulting from the initial abdominal access and are usually related to prior abdominal surgery, previous pelvic inflammatory disease or diverticulitis and severe obesity (103). On the other hand any type of MIS offers better outcomes than laparotomy in terms of a shorter hospital stay, decreased perioperative morbidity, less postoperative pain, and faster recovery. When EOC is treated with MIS, several advantages are available and due to smaller incisions that heal more quickly adjuvant chemotherapy can begin sooner. MIS has been nowadays incorporated in EOC treatment to evaluate optimal debulking surgery with staging laparoscopy and to manage ESOC and advanced stage disease at primary diagnosis and after NACT, and recurrent disease. According to an ovarian cancer patient’s stage, the MIS procedure will depend on the goal of surgery. Although existing studies do not demonstrate deleterious survival effects associated with MIS for ovarian cancer, these data must be viewed with caution given the significant methodological shortcomings in the existing literature and we cannot ignore lesson learned from Laparoscopic Approach to Cervical Cancer trial that showed how MIS in early-stage cervical cancer patients had a detrimental effect (104).

We must be aware that the absence of significant differences both positively and negatively on survival between minimally invasive vs. open approach presented in many studies might be due to the small sample size of the studies not able to detect any statistically significant difference. Therefore, to validate the use of MIS larger prospective studies and the development of future randomized interventional studies are needed in order to identify patients affected by ovarian cancer at different stage who can benefit from a successful minimally invasive approach (40).

## Conclusion

MIS can be proposed in a wide variety of situations characterizing EOC, from staging laparoscopy to treatment of recurrence. Women treated will experience a lower rate of intra and post-operative complications than those undergoing open surgery. It is of crucial importance that all ovarian cancer patients should be treated in a referral high volume cancer centers with surgeons with an intensive training in MIS. It is in fact important to keep in mind that surgical comparative studies are based on the level of each surgeon, so findings cannot be applied equally to all surgeons and to all centers. If these assumptions are met and patient selection is correct, a minimal invasive approach can be a real advantage in any stage EOC treatment.

## Data availability statement

The original contributions presented in the study are included in the article/supplementary material, further inquiries can be directed to the corresponding author.

## Author contributions

MG conceived and wrote the manuscript. GA, DP, GDI, and GC revised the literature and wrote the manuscript. LA revised the manuscript. VDM conceived and revised the manuscript. All authors contributed to the article and approved the submitted version.

## Funding

This study was partially supported by Italian Ministry of Health—Ricerca Corrente Annual Program 2024.

## Conflict of interest

The authors declare that the research was conducted in the absence of any commercial or financial relationships that could be construed as a potential conflict of interest.

## Publisher’s note

All claims expressed in this article are solely those of the authors and do not necessarily represent those of their affiliated organizations, or those of the publisher, the editors and the reviewers. Any product that may be evaluated in this article, or claim that may be made by its manufacturer, is not guaranteed or endorsed by the publisher.
